# Hospital Meetings and Developments

**Published:** 1906-03-10

**Authors:** 


### HOSPITAL MEETINGS AND DEVELOPMENTS.

### THE SEAMEN'S HOSPITAL SOCIETY ("DREADNOUGHT").

### The London Schools of Tropical and Clinical Medicine.

The annual meeting of Governors of this Society was held at the offices in Cockspur Street on February 28. There was a crowded attendance, and the chair was taken by Sir George Vyvyan, Deputy Master of Trinity House, in the unavoidable absence of the Duke of Marlborough, the President.

The Chairman said that the annual report showed the usual lights and shades. It contained a landmark in the annals of the Society, in the shape of the banquet last May when £11,000 was raised. There had been a great falling-off in charitable donations, but this was a complaint that was not confined to their Society alone. After referring in terms of deep regret to the deaths of Sir Frederick Cleeve and Captain W. Ladds, he expressed his great pleasure that Captain Clarke had joined the committee. We in our island home were dependent on merchant seamen for all the necessaries of life. He himself had passed the greater part of his life at sea, and though it had been spent in very well-conducted ships, yet he could speak from his own experience of the ill-treatment of the seamen, amounting to torture in some cases, and of the rough and ready methods of doctoring, "methods of barbarism," he might call them, although well meant. The smaller vessels often made long voyages with sick seamen on board, who when they at length reached the hospital came sometimes too late. All this pointed to one great want and that was that when undergoing examination for a master's certificate men should be required to have a certain amount of medical and surgical knowledge and first-aid. The Society required to be better known, and he suggested to their very able staff that if only they could discover the cure for sea-sickness it would cover them with glory and attract a large amount of public attention to the Society. The hospitals had certain requirements and needed money to be spent on them to bring them up to the present high standard. A new out-patient department was needed, floors wanted renewing, and additional bed accommodation was required, but in the present state of the funds the committee did not feel themselves justified in going to any further expense. The Society had lately widened its scope by the establishment of two schools, the London School of Tropical Medicine and the London School of Clinical Medicine at the "Dreadnought" Hospital. In conclusion, he said it was not too much to hope, nay, even to demand, that those who were in health should assist in supporting an institution that was doing so much service to humanity.

Sir Henry Burdett, in moving the adoption of the report, associated himself with the Chairman's expressions of regret at the death of Sir Frederick Cleeve, with whom he had worked for thirty-two years in conncetion with this hospital. The hospital occupied a unique position among the hospitals of this country. Originally its proud boast was that it was the one institution which admitted patients regardless of race, creed, or colour from all the countries of the world. That was their charter. In discharging their trust the committee began to make new departures so as to bring the means of relief nearer to the sailors for whose benefit the "Dreadnought" was established. They opened dispensaries at Gravesend, near the docks, and close to the Sailors' Home. Then, as there was great need for accommodation at the docks, they started the branch hospital there. When all this good work had been done and the benefit manifested itself not only to the patients, but to their employers and to the public at large there came a great change in public opinion. He thought the Governors owed a debt of gratitude to the committee and to its Chairman, Mr. Percival Nairne, under whose administration the "Dreadnought" had taken a fresh lease of life. It was fortunate also that they had had Mr. Joseph Chamberlain at the Colonial Office, to whom humanity would ever be indebted for having made it possible to establish the London Tropical School of Medicine. That school had started in a small way, but its usefulness was so soon manifested that it was extended. No better ward-unit could be found in any hospital in the world than was to be seen in the branch hospital; a testimony to the wisdom, knowledge, and care of those who had the management of the Society. The fact that the Colonial, Indian, and Foreign Offices and the Army and Navy sent students direct to the Tropical School of Medicine was a proof of its usefulness and efficiency. They had the satisfaction of knowing that certain members of the medical profession had acquired great reputation out of the work they had done there. The diminution of suffering which had already accrued through this school was a monument of which any institution might be proud. The sailor's life, even in these times, was not a happy one in the day of sickness or when struck down by accident. The committee recognised this by opening the London Clinical School of Medicine, where not only diseases and accidents were to be seen, but accidents that had taken place some time before admission, requiring the greatest knowledge and care in treatment. The success of the London Clinical School at Greenwich was well assured from the fact that it had been welcomed by the medical profession and that in consequence it had secured so eminent a staff that any medical school in the world might be proud to possess it. The school must fill a great need in several ways: First, it was an enormous benefit and advantage to the patients; and secondly, it conferred great benefits upon the medical profession who were able to avail themselves of a clinical school, especially as it was affiliated to the special hospitals. It was also of great benefit to the public at large by the training it gave to medical students. This hospital was free from the objection which attached to so many of the London hospitals that they did too much free medical work. The increase in the growth of the hospital population of London was considerable—more than the increase in the growth of the whole population. If people were allowed to consider that whatever their social position, they had a right to claim gratuitous medical relief, the principles of individual honour and effort would be destroyed and the whole population might be pauperised.

The adoption of the report was seconded by Sir Richard Tracey and supported by Sir Patrick Manson, who dwelt upon the enormous advantage it was to the patients for the medical officers to be attended by a band of students. He gave one or two illustrations of the way in which at the London Tropical School of Medicine they had been able, by analysis of the blood to discover the cause of diseases. The 564 students who had passed through the school were all qualified for work in the tropics and would be of immense service to the inhabitants of those regions.

A resolution cordially approving of the action of the committee in establishing at the "Dreadnought" Hospital the London School of Clinical Medicine and affiliating various special hospitals with it was moved by Mr. Percival Nairne, who expressed the thanks of the Society to the Admiralty and to the Thames Ironworks for giving them half of the proceeds from the charge made to visitors on board the new battleship *Dreadnought* amounting to £550. It was always a question for the Society how far they might devote their funds to objects that did not appear to be directly for the benefit of seamen; but it must be borne in mind that it was one of the duties of a hospital to provide for the proper training of nurses and medical officers.

Sir William Bennett, in seconding the resolution, said that this School of Clinical Medicine would have a far-reaching influence beyond the limits of the United Kingdom, and it would become a matter of Imperial importance.

The election of officers was moved by Admiral de Kantzow and seconded by Professor Tanner Hewlett. Sir Frederick Young moved the election of various Hon. Life Governors, and Dr. Rogers seconded the motion. A vote of thanks to the Chairman was moved by Dr. Barnes and seconded by Admiral Sir Robert Harris.

### LONDON TEMPERANCE HOSPITAL.

The annual meeting of this Hospital was held on March 5th at 7 p.m., the Bishop of London presiding. There was a large attendance of governors and sympathisers with the work of the hospital.

The Hon. Secretary (Dr. Dawson Burns), in a few opening remarks, said that the present occasion marked the silver jubilee of the present building, which had been opened by the then Lord Mayor in 1881. He was glad to be able to tell them that Lord Alverstone, Lord Chief Justice of England, had been elected President of the hospital for the coming year. This hospital held a specially unique position as regards the temperance movement; there were many temperance societies, but only one temperance hospital.

The Bishop of London, who was greeted with loud and prolonged cheers, said that it gave him the greatest pleasure to be present that night—first because he had himself been a teetotaller for 23 years and, probably in consequence, had enjoyed uniform good health all that time; second, because in his position he had the opportunity of seeing the terrible effects of drink in his diocese, and he knew well that a hospital like this was a bright star against the background of drink in London; thirdly, because of the declaration made by 2,000 medical men in 1847 that abstinence from alcoholic beverages would greatly contribute to the health, prosperity, morality, and happiness of the human race. There had been a wonderful change of late years in the opinion of the medical profession as regards temperance. Sir Benjamin Richardson had been looked upon as a fanatic in his time. As an instance of the change, one of the leading medical men at the London Hospital had said that if temperance principles were observed they could close one of the wards at the "London." He himself remembered, when visiting his parishioners at that hospital, how many cases were brought there by drink. The statement on page 11 of the report was a very striking one, showing as it did the reduction in alcoholic expenditure at 19 London general hospitals between 1884 and 1904; giving an average diminution from 4s. per patient to 1s. 10d. He never went round a better ordered hospital than the London Temperance. To-day was not the first time he had seen it; he had paid it a surprise visit some two years ago at 10 o'clock at night, and had then found everything in exactly the same working order as on that day. He pleaded with them for increased support for the splendid experiment which had now proved such a success. He was on the Council of King Edward's Hospital Fund, and would watch their interests there. He appealed to them for generous help towards the £10,000 needed for the extension of the hospital, as recognition of the splendid work done there, and he wished Godspeed to the London Temperance Hospital.

A vote of thanks to all "engaged in the work of this hospital, which, besides its utility as a general hospital, is furnishing the medical profession at large with valuable evidence on the disuse of alcohol in the satisfactory treatment of disease," was moved by the Rev. Canon Fleming, who said that he had been a friend of the hospital from the beginning, and held that every total abstainer ought to support it. He himself had been an abstainer for 40 years.

The vote was seconded by Lady Hope and supported by Mr. Leif Jones, M.P., who remarked that it was exceedingly interesting to observe that as knowledge and science spread the use of alcohol diminished. A committee of medical men some two years ago sent a petition to the Board of Education asking that the truth about intoxicating drinks might be taught in the schools. The matter had been taken up by the Education Authorities, and the suggestion was now being carried out.

At this stage in the meeting the Bishop of London was obliged to vacate the chair, having signified his desire to contribute £10 towards the extension scheme. The chair was then taken by Sir T. Vezey Strong, Chairman of the Board of Management.

Sir William Collins, F.R.C.S., M.P., in replying to the vote of thanks, said that he had a very strong affection for the hospital, with which he had been connected for 18 years, and during that time 10,000 in-patients had passed through his hands, and 4,500 operations had been recorded in the surgical department. Their percentage of recoveries compared favourably with that of any other general hospital in London. The surgical side of the hospital was in a very satisfactory and flourishing condition. He congratulated Mr. Paterson, their assistant surgeon, on having recently been made Hunterian Lecturer at the Royal College of Surgeons. They had a registrar and chloroformist who was second to none. He could not speak too highly of the work done by the Matron. The new out-patients' department was shortly to be started, and the Council of King Edward's Hospital Fund were very anxious that there should be no further delay about it. A museum and laboratory would also be attached to it. As the subscribers were probably aware, they had an aseptic ward which had served as a model in many other hospitals, and the London Temperance had been the pioneer in this country by the institution of this ward. It was a privilege and a pleasure to lecture to the nurses at that hospital, and the results of their examination were excellent. They badly needed a convalescent home in the country, where the cure of the patients could be carried out on the same lines of treatment. The children's ward was to be improved by having the walls tiled.

Dr. Porter Parkinson stated, as a proof of the popularity of the hospital, that patients came to it again and again. It was also popular with members of the medical profession, especially from the Continent and America, many of whom had gone round the wards. It was a pity the hospital was not a teaching hospital; a very little extra accommodation was needed to bring it up to the required standard of 100 beds.

Mr. Paterson, who followed, urged the provision of a pathological laboratory, which he stated to be a real necessity, and the cost of which would not exceed £800.

Sir T. Vezey Strong said the Board of Management had decided not to undertake any work beyond what was absolutely imperative, and therefore the laboratory, etc., must be held over for the present. In order to accommodate the large number of out-patients they had decided they must have a new out-patients' department, and the specifications for it were now in hand. Only the ground floor and basement would be erected, and it would have a flat roof to keep out the weather. Even for this they would require £10,000. The expenditure per head went steadily down, not by depriv ing the patients of any comforts or necessaries, but by ever-increasing care in buying supplies, and superintendence. Of each pound subscribed 19s. 6d. went to the benefit of the patient and 6d. to provide costs of administration.

A resolution appealing for increased financial support was moved by the Treasurer (Mr. H. Holloway), who said that their expenditure was over £10,000 a year, which was a very small sum compared with the amount of work done. The resolution having been seconded by Dr. Robinson Souttar, the proceedings terminated with a vote of thanks to the Chairman.

### ROYAL NATIONAL ORTHOPÆDIC HOSPITAL.

It is pleasant to note that the speakers at the annual meeting fully recognised the importance of the scheme of amalgamation for the orthopædic hospitals of London which the public owe to the foresight and influence of the managers of the King's Hospital Fund. It will be an immense service to have secured one great, efficient, and perfected orthopædic hospital for the metropolis.

Lord Denbigh, the Chairman of the General Committee, presided on the 28th ultimo at the first annual general meeting of the amalgamated Royal and National Orthopædic Hospitals, held on the premises in Great Portland Street. In moving the adoption of the report and the accounts of the two hospitals merged together as one institution, he said that the completion of the amalgamation was a matter of very considerable importance, and one on which everybody concerned could be congratulated. The King's Hospital Fund Committee had pointed out that it was desirable that as many as possible of the special hospitals should be amalgamated, and after prolonged negotiations that had been carried into effect in their case, and they might now hope for a period of prolonged usefulness for the joint undertaking. One of the most difficult questions which presented itself was the arrangement of the finances. The Royal had most money, but the National had the necessary site for building. However, that matter had been satisfactorily adjusted, and they were much indebted to Mr. Young, of Messrs. Turquand, Young and Co., for the immense amount of trouble he had taken to bring about that result. Plans had been submitted with a view of the erection on that site of a thoroughly efficient hospital, with every modern requirement, which would be of the greatest service to the suffering poor and more especially to the children. He congratulated the medical staff on the care and attention they gave so freely, and on the wonderful cures they brought about. They regretted very much the retirement of Miss Hole, their matron, but she had come to the conclusion that her health would not allow her to continue her duties. He wished to express the gratitude of the committee to their Majesties the King and Queen for graciously extending their patronage to the amalgamated institution as to the old Royal Orthopædic. In conclusion, he expressed the earnest hope that everything possible would be done to obtain the requisite financial support for the great work of rebuilding.

Sir Richard Biddulph Martin, one of the treasurers, seconded the resolution, which was adopted. The committee was re-elected, with the addition of Mr. Luther Martin, and votes of thanks concluded the meeting.

### CITY OF LONDON LYING-IN HOSPITAL.

The 155th annual meeting of the City of London Lying-in Hospital was held on February 21, Mr. George Berry presiding. In moving the adoption of the report and accounts he said that although, in consequence of the rebuilding operations in progress, they were met under not very agreeable conditions, they hoped soon, with the exercise of patience, to be in possession of much more commodious premises. Even then, however, he doubted if they would be able to present the governors with a better report than that now before them. Despite the difficulties under which the work had been carried on, they had had among their 611 in-patients not a single maternal death due to causes connected with childbirth; the only death of a mother in the hospital during the year being from long-standing phthisis, she having been received in a dying condition from that disease. It was obvious that the greatest credit was due to the management—to the Secretary, Mr. R. A. Owthwaite, the matron, and the officers. He thought their new building would cost about £40,000, and when complete it would be a credit to the city of London and a monument to their 150 years of good work. They would all share his regret at the death of their architect, Mr. H. H. Collins, before the completion of the building, but it would remain as a memorial to him.

Mr. Francis, in seconding the motion, referred to the fact that no less than 65,500 children had been born within their walls; while during the past 30 years 45,596 mothers had been attended in their own homes. The building of the new premises was going on successfully, and they hoped it would be completed early in the autumn.

The report was adopted, and also resolutions of thanks to the medical staff and the Committee of Management.

### CENTRAL LONDON OPHTHALMIC.

Mr. Charles Wollaston presided on February 22 at the 62nd annual meeting of the Central London Ophthalmic Hospital. In moving the adoption of the report he said that during 1905 they had treated 12,890 new cases, as compared with 12,820 in the previous year, the number of attendances being 30,488, as compared with 31,114. The in-patients numbered 352, as against 369; accidents and urgent cases, 410, compared with 368; and there were 1,101 operations performed, 303 being of a serious nature. The falling-off in the in-patients was due to the closing of the hospital for two months for disinfection and repainting consequent on an outbreak of scarlet fever. The expenditure of the year had followed normal lines, except that the closing of the wards had allowed of some savings to set off against the extra cost of £80 for painting, etc. They had actually spent £20 less than they had received, but must not forget that they had to pay £280 incurred in the previous year. Toward the Building Fund they had a grant from King Edward's Fund of £150; and both that body and the Sunday Fund strongly urged on them the necessity for rebuilding. During the year, in May last, they had a visit from their patron, Princess Frederica, who expressed herself as delighted with all she saw. On the question of safety from fire, the Committee had consulted Messrs. Merryweather, and on their recommendation had put in hand-pumps and buckets on each floor; and, what he thought was even more important, an iron staircase outside. The electric light had now been put into all wards and rooms not already lit by it; and this had been done at the expense of Mr. Ernest Clarke, one of their honorary staff. As to the question of rebuilding, it was unnecessary for him to reiterate that it had become an absolute necessity. Their fund, however, was only now £7,250, including a legacy of £500 from the late Mr. Buckwell, received since the close of the year; and they did not feel justified in commencing operations till they had £12,000 at least in hand. Their Secretary had been most painstaking and successful in this matter, the fund having increased by £4,000 since his appointment, and he hoped it would not be long before they were able to make a start.

The report having been adopted, an alteration in the rules was made, whereby assistant surgeons of five years' standing would become surgeons. Thanks were accorded to the staff, Committee, and officers, and Mr. Brittin Archer having again urged the desirability of the institution of a Ladies' Guild and the necessity for a resolute effort to induce some wealthy people to help them to put up the urgently needed new premises, the meeting separated with a vote of thanks to the Chairman.

### ST. MARY'S HOSPITAL FOR WOMEN AND CHILDREN, PLAISTOW.

The annual meeting of governors was held on February 27. The chair was taken by the Bishop of Barking, who, after the annual report had been read, said that the figures in the report referring to numbers of patients and attendances, etc., showed general increase. The only unsatisfactory clause was the one dealing with the severe pressure upon the hospital accommodation. It could not but be unsatisfactory to feel that the hospital could not take in urgent and sometimes dying cases. The extension must be pressed on at the earliest possible opportunity. It was a great thing to have collected so much as £7,200; they only wanted now about £2,000 more to enable them to start upon the first section of the work. They wanted further accommodation at the hospital, not only for the patients, but for the staff as well.

The Rev. T. Given-Wilson said that two legacies had been left to him to apply as he thought fit—one for £100 and the second for £200—and he purposed devoting both to the needs of the hospital. He wished to emphasise in the strongest possible manner the great pressure upon the hospital and medical staff. He believed if they could only bring the real wants of the institution before the charitable public they would get the money in a week. As an instance of the severity of the pressure upon the accommodation of the hospital he stated that on occasions children had had to be put into baths instead of beds, in order to avoid turning them away. He had been able to purchase the site for the extension and present it to the hospital by means of a yearly sale of old clothes in his parish, by which they raised £1,000 yearly.

The report and balance-sheet having been passed, a resolution was carried, stating that before Easter a letter should be published in the papers, to be called "The Bishop of Barking's Appeal," to be signed by the Bishop, as Chairman of the Committee, making an appeal for funds for the extension.

### ROYAL WESTMINSTER OPHTHALMIC HOSPITAL.

The annual meeting was held on March 2, when Sir Charles Turner, K.C.I.E., Chairman of the committee of management, presided.

The adoption of the report was moved by the Chairman, who said that the hospital had been well supported by the public during the past year, and they had been able to close the year with a small balance to the good. The net increase of subscriptions, donations, and other receipts had been £93 12s. A still more gratifying feature was the testimony received from the patients as to their satisfaction at the arrangements made for their care and comfort. The whole expenditure was slightly less than £2,500 for the year, which the donations made by the patients in the various boxes was over £250, so that one-tenth of the expenditure was contributed by those for whose benefit the hospital was maintained. In answer to the suggestion that those attending the hospital might have no right to free treatment, he replied that this year they had made out a very elaborate return, showing the classes of the patients received there, by which they could be assured that the patients were precisely those who were obliged to have recourse to charitable institutions to obtain the best medical care and attention. There were large numbers of servants, shop-assistants, etc., and they came not only from London, but from all parts of the country. The total number of out-patients treated during 1905 was 10,747. The returns showing the different counties from which the patients came demonstrated the fact that the hospital, no doubt owing to its central position, was able to afford assistance to many sufferers from the provinces who came to London for medical relief. King Edward's Hospital Fund had come to their assistance in a very acceptable manner. They had incurred a debt of about £9,000 by the remodelling of the hospital. To meet this they had taken a certain sum from their sustentation fund, leaving a total of £5,200. Towards this King Edward's Hospital Fund had granted them altogether £1,400, and they very much hoped by the end of this year the whole debt, which now only stood at £400, would be wiped out. The cost of each in-patient averaged only 23s. per week, and that of each out-patient 1s. 7¼d. After taking into consideration the fact that the patients in an ophthalmic hospital were not so costly as those in a general hospital, yet the figures would compare most favourably with those of any other similar institution.

A peculiar feature of this hospital had always been its medical school. Dr. Guthrie, their founder, an officer in the medical service of the East India Company, had founded it in order that it might be a school for the officers of the Company, who had to go to and return from India in charge of troops; amongst those troops ophthalmia was extremely prevalent, and Dr. Guthrie, in order that his brother-officers might be thoroughly well educated in ophthalmic surgery, instituted this hospital. It had always been in close connection with the Services. It had been said that it was not right to raise money for the hospitals and then spend a portion of it upon medical schools. No one need be deterred from subscribing to this hospital by the fear that any portion of the money would be spent on the medical school. The surgeons of this hospital relieved the committee of practically all expense connected with the medical school. They completely furnished the operating theatre when the hospital was remodelled, established and maintained the library, and defrayed whatever other expenses were incurred by the medical school. They were indebted to the surgeons for being able to maintain an efficient school of ophthalmic surgery without expense. Under the will of Mr. I. Cone, the hospital would obtain the sum of £10,000, which would more than replace the sum taken from the sustentation fund for rebuilding. They had made a practice for some years past of setting aside their legacies, whenever not immediately required for any very urgent purpose, in order to augment the sustentation fund, thinking in this way to fulfil the wishes of the donors. As the result of this practice they had an annual income of over £600 from this fund. The one circumstance which caused them deep regret in the past year was the fact that their late Secretary, Mr. Thomas Beattie Campbell, having been attacked by paralysis, had ceased to be able to perform the duties he had discharged so faithfully for a great many years. It was owing to his good work that the hospital took the position it now occupied. The committee had decided to grant him a small pension.

The resolution was seconded by the Hon. Arthur Brodrick, who joined in the Chairman's expressions of regret at the loss of Mr. Campbell's services.

Various votes of thanks were then passed, and the proceedings terminated.

